# Ongoing disease-activity and change in ILAR categories in a Nordic juvenile idiopathic arthritis cohort

**DOI:** 10.1186/1546-0096-10-S1-A41

**Published:** 2012-07-13

**Authors:** Ellen Nordal, Marek Zak, Lillemor Berntson, Kristiina Aalto, Suvi Peltoniemi, Susan Nielsen, Troels Herlin, Pekka Lahdenne, Bjørn Straume, Anders Fasth, Marite Rygg

**Affiliations:** 1 Århus University Hospital, Arhus, Denmark; 2 Children's Hospital, Helsinki University Hospital, Helsinki, Finland; 3 Norwegian University of Science and Technology, Tronsheim, Norway; 4 Rigshospitalet, Copenhagen, Denmark; 5 The Finnish Medical Society Duodecim, Helsinki, Finland; 6 University Hospital of North Norway, Tromso, Norway; 7 University of Gothenburg, Gothenburg, Sweden; 8 University of Helsinki, Helsinki, Finland; 9 University of Tromsø, Tromso, Norway; 10 Uppsala University Children's Hospital, Uppsala, Sweden

## Purpose

Juvenile idiopathic arthritis (JIA) is the most common rheumatic disease in children. During the last decade international consensus on the ILAR (International League Against Rheumatism) classification of this heterogeneous group of chronic childhood arthritis has been reached. In an era of modern medical treatment, epidemiologic studies on outcome in unselected cohorts of children with JIA are important. The aim of the study was to describe disease characteristics, ILAR categories and remission status during the first eight years after disease onset in a cohort of Nordic children with JIA in a population-based setting.

## Methods

Consecutive cases of JIA from defined geographical areas of Denmark, Finland, Sweden and Norway with disease onset in 1997 to 2000 were included. The incidence of JIA in the study area in 1997 - 98 was 15 per 100 000/ year. The study aimed to be as close to population-based as possible. JIA categories were determined according to the ILAR criteria at onset and during disease course up to ten years after onset. Clinical data and disease activity were registered by a set protocol at regular follow-up visits.

## Results

Of 500 included children, 440 (88%) had a follow-up visit seven years or more after disease onset (median 96 months, range 84-147). The number of visits varied between 2 -10 (median 5). Among the 440 children followed, 66% were female, median age at onset was 6 years and 51% were oligoarticular at 6 months. Extended disease course with more than four joints involved developed in 34.8% of the children with oligoarticular onset. During the disease course, additional changes in ILAR categories were seen in 10.5% of the children. Uveitis developed in 88 (20%). Synthetic and/or biologic DMARDs were reported to be used in 254 (58%) children during the disease course. At the last follow-up, 42% of the children were in remission off medication, 9% were in remission on medication and 49% were not in remission.

## Conclusion

In this prospective, longitudinal study of JIA in a population-based setting, ongoing disease was evident in a majority of the children, and changes in ILAR categories occurred during disease course. The present results underline the need to identify early predictors of outcome, to further improve therapy and to continue long-term follow-up of children with JIA.

## Disclosure

Ellen Nordal: None; Marek Zak: None; Lillemor Berntson: None; Kristiina Aalto: None; Suvi Peltoniemi: None; Susan Nielsen: None; Troels Herlin: None; Pekka Lahdenne: None; Bjørn Straume: None; Anders Fasth: None; Marite Rygg: None.

